# Environmental Hot Spots and Resistance-Associated Application Practices for Azole-Resistant *Aspergillus fumigatus*, Denmark, 2020–2023

**DOI:** 10.3201/eid3008.240096

**Published:** 2024-08

**Authors:** Maiken Cavling Arendrup, Rasmus Krøger Hare, Karin Meinike Jørgensen, Ulla E. Bollmann, Tina B. Bech, Cecilie Cetti Hansen, Thies M. Heick, Lise Nistrup Jørgensen

**Affiliations:** Rigshospitalet, Copenhagen University, Copenhagen, Denmark (M.C. Arendrup);; Statens Serum Institut, Copenhagen (M.C. Arendrup, R.K. Hare, K.M. Jørgensen);; Geological Survey of Denmark and Greenland, Copenhagen (U.E. Bollmann, T.B. Bech, C.C. Hansen);; Aarhus University, Flakkebjerg, Slagelse, Denmark (T.M. Heick, L.N. Jørgensen)

**Keywords:** Aspergillus fumigatus, azole fungicide, azole resistance, agriculture, horticulture, aspergillosis, compost, manure heap, flower bed, *A. fumigatus*, ARAF, fungi, microsatellite typing, Denmark

## Abstract

Azole-resistant *Aspergillus fumigatus* (AR*Af*) fungi have been found inconsistently in the environment in Denmark since 2010. During 2018–2020, nationwide surveillance of clinical *A. fumigatus* fungi reported environmental TR_34_/L98H or TR_46_/Y121F/T289A resistance mutations in 3.6% of isolates, prompting environmental sampling for AR*Af* and azole fungicides and investigation for selection of AR*Af* in field and microcosmos experiments. AR*Af* was ubiquitous (20% of 366 samples; 16% TR_34_/L98H- and 4% TR_46_/Y121F/T289A-related mechanisms), constituting 4.2% of 4,538 *A. fumigatus* isolates. The highest proportions were in flower- and compost-related samples but were not correlated with azole-fungicide application concentrations. Genotyping showed clustering of tandem repeat–related AR*Af* and overlaps with clinical isolates in Denmark. *A. fumigatus* fungi grew poorly in the field experiment with no postapplication change in AR*Af* proportions. However, in microcosmos experiments, a sustained complete (tebuconazole) or partial (prothioconazole) inhibition against wild-type *A. fumigatus* but not AR*Af* indicated that, under some conditions, azole fungicides may favor growth of AR*Af* in soil.

Azole resistance in *Aspergillus* *fumigatus* fungi has increased during the past 25 years. Increasing evidence documents that selection of azole-resistant *A. fumigatus* (AR*Af*) takes place in the environment (*1*,*2*). Investigations have been performed or initiated in several countries to investigate the relative contributions of various environmental azole fungicide applications to selection for AR*Af* (*3*–*5*).

In Denmark during June–August 2009, AR*Af* was first found in 1/17 *A. fumigatus* isolates from hospital surroundings and 3/21 from a park in Copenhagen (*6*), but subsequent environmental soil and air samples collected during September–October 2013 were negative for AR*Af* (*7*). That finding is somewhat in contrast to findings in clinical samples from Denmark. After the first isolation of TR_34_/L98H mutants in late 2007 and TR_46_/Y121F/T289A in 2012 (*7*–*9*), an increasing rate of AR*Af* of environmental origin from 1.5% (2/133) in 2007–2009 to 3.6% (5/137) in 2018 has been found in patients with cystic fibrosis (*8*,*10*). Moreover, during 2018–2020, the nationwide surveillance of AR*Af* revealed a rate of 3.6% environmental AR*Af* among 1,083 patients (*11*).

Which environmental azole fungicide uses are potentially safe and which contribute mostly to the increasing proportion of AR*Af* is not clear. However, because selection of resistance through either emergence of resistance in a susceptible isolate or favored growth of an already existing AR*Af* subpopulation requires *A. fumigatus* multiplication, azole residues in soils or plant debris where *A. fumigatus* fungi thrives are probably the biggest source for dissemination of AR*Af*. Prior studies have suggested that hot spots for AR*Af* include azole-treated flower bulb production (*1*), plant waste piles, and composting heaps (*1*,*12*), whereas cold spots probably include animal manure and grain (*1*,*13*) and arable farming (*14*,*15*), including potato fields (*3*). However, variable findings have been reported for several settings, including greenhouses and strawberry crops (*3*,*5*,*16*).

On the basis of those findings, the Danish Ministry of Environment supported a research project about the presence and selection of AR*Af* in Denmark. The project included extensive environmental sampling with determination of azole-susceptible and -resistant *A. fumigatus* and of azole concentrations; characterization of resistance mechanisms and molecular genotypes to determine if resistant genotypes come from outside (by wind and goods) or multiply and expand in Denmark; and microcosmos and field experiments investigating the potential of various azole fungicides to select for AR*Af*.

## Materials and Methods

### Environmental Hot Spot and Field-Experiment Sampling

We collected 366 samples (Appendix Table 1): agricultural fields (air and soil; n = 167, including 40 samples obtained before/between/after azole spraying); park and private garden soil (n = 60); flower and potatoes (n = 100); compost soil (from garden waste) and compost heaps from vegetable waste and garden waste (n = 20); animal manure heaps with straw or peat and associated stable bedding (n = 25); and wood paint–associated soil (n = 14). We sampled air (1 m^3^/sample) through a gelatin filter by using a Sartorius MD8 Airport Portable Sampler (https://shop.sartorius.com). We placed the gelatin filter on yeast glucose chloramphenicol (YGC) agar and incubated it 1 day at 37°C, 1 day at 50°C, and 1 day at 37°C, inspecting it daily. That procedure favored growth of *A. fumigatus* fungi over other molds, thereby enhancing *A. fumigatus* isolation in a pilot study. Solid samples (e.g., soil top 5 cm [*5*], compost, manure heap) were suspended in sterile water with 0.1% Tween 20 (2.5 mL/g sample), vortexed, and allowed to settle for 10–15 minutes. We transferred ≈10 mL top fluid to a new tube, vortexed it, and cultured 500 µL or 250 µL on YGC and on YGC supplemented with tebuconazole (3 mg/g agar [YGC-Teb]). We centrifuged the remaining fluid (3,000 rpm, ≈1,942 g, 10 minutes), discarded ≈8 mL supernatant, and resuspended the pellet in the remaining liquid followed by plating of 500 µL on YGC and YGC-Teb. For air samples, we incubated all plates as described above.

We isolated *A. fumigatus* fungi (maximum 30 isolates/sample), subcultured, and identified by using macro- and micro-morphology and thermotolerance of 50°C supplemented with matrix-assisted laser desorption/ionization time-of-flight mass spectrometry (Bruker, https://www.bruker.com) and the online available spectrum database mass spectrometry imaging when needed (*17*,*18*). When we identified mixed TR_34_/L98H and TR_46_/Y121F/T289A mutations, we attempted isolation from susceptibility plate wells containing voriconazole (favoring TR_46_/Y121F/T289A) and posaconazole (favoring TR_34_/L98H).

### Susceptibility Testing

Initially, *A. fumigatus* colonies on YGC-Teb underwent azole-resistance screening (EUCAST E.Def 10.1), followed by determination of MICs of itraconazole, posaconazole, isavuconazole, and voriconazole (EUCAST E.Def 9.3) if screening positive. Because of equal performance of YGC-TEB and E.Def 10.1, we subsequently omitted the E.Def 10.1 screening step (*19*,*20*). We compared individual proportions of AR*Af* pairwise by using a χ^2^ or Fisher exact test with the GraphPad Prism 9.3.1 program (https://www.graphpad.com).

### Extraction and Concentration Determination of Azoles

We analyzed azole content as previously described for soil samples by using sonication/shaking-extraction and high-performance liquid chromatography–tandem mass spectrometry analysis (*21*) with minor modifications: soil samples were sieved (2 mm) and homogenized manually; potted plant soil/root mix and freeze-dried potato peels were homogenized in a blender (Appendix Table 2). We prepared blank and control samples as well as calibration standards in a reference matrix (organically farmed soil or potato peel), extracted, and analyzed together with each set of samples. When no matching reference matrix was available (potted plants, compost), we used standard addition.

### Molecular Characterization of Azole Resistance Mechanisms

We sequenced the *cyp51A* gene, including promoter, as previously described for AR*Af* isolates and selected susceptible *A. fumigatus* isolates (*8*,*22*) (Appendix Table 3). Azole-resistant isolates that were *cyp51A* wild-type underwent full-length *hmg1* sequencing as previously reported (*23*), with some modifications (Appendix Table 4). We assembled sequences and compared them with appropriate reference sequences (*cyp51A*, GenBank accession no. AF338659; *hmg1*, GenBank accession no. Afu2g03700) by using CLC Main Workbench versions 22 and 23 (QIAGEN, https://www.qiagen.com). We reported only tandem repeats in the promoter region (*cyp51A* only) and mutations leading to amino acid changes.

### Genotyping

We conducted genotyping by using the short tandem repeat *A. fumigatus* (STR*Af*) method with all 9 microsatellite markers as previously described (*24*) (Appendix Table 5). We performed genotype analyses by using BioNumerics versions 7 and 8 (bioMérieux, https://www.biomerieux.com), illustrated as minimum spanning trees with default settings. We compared the genotype to worldwide genotypes from the Czech Republic (n = 1), Australia (n = 2), China (n = 8), the United Kingdom (n = 10), Cuba (n = 14), Switzerland (n = 71), Germany (n = 100), the United States (n = 102), Belgium (n = 108), Norway (n = 209), Spain (n = 219), and the Netherlands (n = 615) (*9*), as well as addition genotypes not previously reported from Finland (n = 1), Austria (n = 3), and Sweden (n = 5).

### Microcosmos Selection Experiments

For microcosmos experiments, we placed 4 g dry sterile soil and 1 mL of 2–5 × 10^2^ CFU/mL *A. fumigatus* solution (wild type, TR_34_/L98H, and TR_46_/Y121F/T289A) in 0.85% NaCl in 25 mL glass vials. We included sandy soil (total organic carbon content 0.92%) and a soil with high organic content (total organic carbon content 5.68%). The soils originated from fields organically farmed for 40 years (Svanholm Gods, Denmark). The microcosmos vials were initially incubated at 10°C, 15°C, and 20°C and consecutively sampled for *A. fumigatus* and AR*Af* quantification (Appendix Figure 1). For selection experiments, we chose incubation at 20°C and added 100 µL azole fungicide solution (tebuconazole [Folicur EW-250, 250 g/L; Bayer]), prothioconazole [Proline EC-250, 250 g/L; Bayer], mefentrifluconazole [Revysol, 100 g/L; BASF, https://agriculture.basf.com], or MilliQ water [control; Sigma Aldrich, https://www.sigmaaldrich.com]) 2 days after inoculation in application concentrations of 2.5–2,500 mg/L and homogenized the content with an inoculation loop. Final wet-weight concentrations were 0.049–49 mg/kg (spike solution concentration × applied volume)/dry weight) (Appendix Table 6).

### *A. fumigatus* and AR*Af* Quantification in Microcosmos by PCR

We extracted DNA from the microcosmos samples (≈250 mg) and the collected soil samples by using DNeasy PowerLyzer PowerSoil Kit (QIAGEN) and 50 μL elution buffer. To quantify, we used quantitative PCR or droplet digital PCR (Appendix Table 7). For the first microcosmos experiments, the target was a multicopy internal transcribed spacer, and for subsequent experiments, we used primers and probes targeting the *cyp51A* promoter able to distinguish TR_34_/L98H and TR_46_/Y121F/T289A (Appendix Table 6). We ran controls for the standard curve and samples in triplicate.

## Results

### Environmental Sampling

Environmental sampling consisted of 366 samples and 4,538 *A. fumigatus* isolates (Table 1). In 2020, AR*Af* harboring TR_34_/L98H or associated variants (TR_34_/T-67G/L98H or TR_34_/L98H/S297T/F495I), specifically, were found in all sample types and years, except 1 potato field. In 2021 and 2022, *A. fumigatus* fungi harboring TR_46_/Y121F/T289A or associated variants (TR_46_/Y121F/T289A/S363P/I364V/G448S or TR_46_**^3^**/Y121F/M172I/T289A/G448S) were found in samples from fields, flowers/flower beds, compost, and stable bedding.

**Table 1:** Overview of *Aspergillus fumigatus* and AR*Af* showing total and TR_34_/L98H-related [TR_34_] and TR_46_/Y121F/T289A-related [TR_46_] isolates from the environment, Denmark, 2020–2022*

Location (samples/sites), date	Samples, no. (%)					Isolates of *Af* and AR*Af*			
	*A. fumigatus*	AR*Af*	TR_34_	TR_46_		*Af*, no. (no./sample)	AR*Af*, no. (%)	TR_34_, no. (%)	TR_46,_ no. (%)
Field soil (84/7)									
Cereal and potato (44/5), 2020	43 (98)	1 (2.3)	1 (2.3)	2		318 (7.2)	1 (0.3)	1 (0.3)	0
Cereal (40/2), 2022 May–Sep	40	8 (20)	5 (13)	1 (3)		360 (9.0)	10 (2.8)	7 (1.9)	1 (0.3)
Field air (63/3)									
Field air (26/1), 2020	23 (100)	2 (7.7)	2 (7.7)	0		181 (7.0)	3 (1.7)	3 (1.7)	0
Field air (37/2), 2021	35 (95)	8 (21.6)	5 (13.5)	1 (2.7)		273 (7.4)	18 (6.6)	14 (5.1)	1 (0.4)
Vegetables (40/10), 2020									
Potato-supermarkets (24/6)	7 (29)	0	0	0		9 (0.4)	0	0	0
Potato-farm shop (8/2)	8 (100)	2 (25)	2 (25)	0		19 (2.4)	2 (10.5)	2 (10.5)	0
Potato-field (Flakkebjerg) (8/2)	8 (100)	2 (25)	2 (25)	0		127 (15.9)	4 (3.1)	4 (3.1)	0
Flower-producers soil (50/3), 2020 and 2021									
Poinsettia (20/2), Campanula (10/1), 2020	30 (100)	8 (27)	6 (20)	0		516 (17.2)	13 (2.5)	11 (2.1)	0
Cactus (10/1), 2020	10 (100)	0	0	0		200 (20.0)	0	0	0
Poinsettia (10/1), 2021	10 (100)	2 (20)	2 (20)	1 (10)		289 (28.9)	14† (4.8)	10 (3.5)	3 (1.0)
Flower-producers air (10/1), 2021	9 (90)	0	0	0		24 (2.4)	0	0	0
Park & garden flowerbed soil (60/5), 2021	59 (98)	18 (30)	14 (23.3)	4 (6.7)		1,476 (24.6)	52 (3.5)	44 (3.0)	8 (0.5)
Allotment near soil (14/14), 2021									
Allotment houses (14)†	14 (100)	2 (14.3)	2 (14.3)	0		358 (25.6)	4† (1.1)	3 (0.8)	0
Compost related (20/3), 2022									
Recycle soil from garden waste (5)	5 (100)	4 (80)	4 (80)	0		219 (43.8)	6 (2.7)	6 (2.7)	0
Compost heap garden waste (10)	10 (100)‡	5 (NP)	5 (NP)	4 (NP)		12‡ (100)	11 (92)	7 (58)	3 (25)
Compost heap vegetable production (5)	5 (100)	5 (100)	5 (100)	3 (60)		21§ (100)	21 (100)	17 (81)	4 (19)
Manure heaps from horses (12/2), 2022									
Center 1 (7), 2022 Feb	5	3 (43)	3 (43)	0		54 ((7.7)	14 (25.9)	14 (25.9)	0
Center 2 (5), 2022 Nov	3	0	0	0		19 (3.8)	0	0	0
Horse stable and beddings (13/1), 2022									
Stable bedding with wheat (2)	2	0	0	0		8	0	0	0
Stable bedding with barley (3)	3	2	1	1		33 (11)	13 (39)	12 (36)	1 (3)
Stable bedding with peat (2)	1	1	1	0		5 (2.5)	2 (40)	2 (40)	0
Fresh wheat (2)	2	0	0	0		10	0	0	0
Fresh barley (2)	1	0	0	0		5	0	0	0
Fresh peat (2)	1	0	0	0		2	0	0	0
Total (366)	334 (91.0)	73 (20.0)	60 (16.0)	15 (0.04)		4,538 (12.4)	188 (4.2)	157 (3.5)	21 (0.5)

*Darker red indicates increasing percentage. AR*Af*, azole-resistant *A. fumigatus, *NP, not possible to determine exact denominator because of uncountable number of colonies on the plate. †One AR*Af* from poinsettia harbored an Hmg1 F262-deletion and 1 from painted wood–related soil harbored an Hmg1 E306K alteration within the sterol- sensing domain. ‡Plates were massively overgrown by Mucorales spp. From 5 samples, it was possible to perform *A. fumigatus* PCR and direct target gene sequencing yielding TR_34_/L98H, TR_46_/Y121F/T289A, or both. §Of >200 resistant colonies per sample (growing on tebuconazole containing agars), 21 individual colonies were selected for susceptibility testing and target gene sequencing.

#### Agricultural Fields

AR*Af* was less common during 2020 (2.3%–7.7% of soil and air samples and 0.3%–1.7% of isolates) than during 2021–2022 (20%–21.6% of soil and air samples and 2.8%–6.6% of isolates). Most AR*Af* harbored TR_34_/L98H (25/32, 78%), whereas 1 harbored TR_46_/Y121F/T289A and 1 harbored TR_46_/Y121F/T289A/S363P/I364V/G448S (6% of AR*Af*). Air sampling was performed before (15 samples), during (29 samples), and after (19 samples) harvesting. The *A. fumigatus* counts were highest in samples taken during harvest (380 [13.1/sample]), compared with before harvest (28 [1.9/sample)] and after harvest (46 [2.4/sample]). Ten air samples (10/63 [15.9%]) contained AR*Af*, 8 of which were taken during harvest (8/29 [27.6%]). Among 454 *A. fumigatus* air isolates, 4.6% were AR*Af* (including 3.7% TR_34_/L98H and 0.2% TR_46_/Y121F/T289A).

#### Produce

Potatoes from supermarket potatoes (washed and bagged) contained very little *A. fumigatus* and no AR*Af* (Table 1). Potatoes from the farm shop and fields had some soil on the surface. All potato samples were positive for *A. fumigatus* fungi (2.4–15.9/sample), and 25% (4/16) samples contained *ARAf* harboring TR_34_/L98H (3.1%–10.5% of isolates). Flowerpot soil samples from 3 flower types and nurseries contained high amounts of *A. fumigatus* fungi. AR*Af* was absent in cactus pot soil, whereas 25% (10/40) of samples from poinsettia and campanula contained AR*Af* (2.5%–4.8% of isolates), including TR_34_/L98H or TR_34_/L98H/S297T/F495I (21/27 AR*Af* isolates during 2020–2021) and TR_46_/Y121F/T289A or TR_46_/Y121F/T289A/S363P/I364V/G448S (3/14 AR*Af* isolates during 2021). One AR*Af* harbored an F262 deletion within the sterol-sensing domain of Hmg1, which has previously been associated with azole MIC elevation (*23*). Last, air samples from a plant nursery contained few *A. fumigatus* fungi and no AR*Af*.

#### Flower beds

From flower beds sampled in 3 public parks and 2 private gardens, 59/60 samples contained *A. fumigatus* isolates (mean 24.6 isolates/sample). AR*Af* was found at all sites and in 30% of samples, ranging from 5% (1/20) to 47% (7/15) among public parks and 50% (5/10) of samples from private gardens. TR_34_/L98H and TR_34_/T-67G/L98H were found in 85% of AR*Af* isolates and 3% of *A. fumigatus* isolates. TR_46_/Y121F/T289A, TR_46_/Y121F/T289A/S363P/I364V/G448S, and TR_46_**^3^**/Y121F/M172I/T289A/G448S found in 1 park and both gardens constituted 0.5% of *A. fumigatus* isolates and accounted for most AR*Af* (6/7 AR*Af* isolates) in the 2 private gardens.

#### Soil

Soil near painted allotment houses/terraces was sampled because runoff water from painted surfaces might contain azoles. All samples contained *A. fumigatus* isolates (mean 25.6 isolates/sample). Two samples were positive for AR*Af* (14.3% samples and 1.1% *A. fumigatus* isolates); 3/4 AR*Af* isolates harbored TR_34_/L98H, and 1 harbored an Hmg1 alteration E306K in the sterol-sensing domain.

#### Compost

All compost soil samples contained *A. fumigatus* isolates (mean 43.8 isolates/sample), and 4/5 samples contained AR*Af* isolates harboring TR_34_/L98H (2.7% of isolates). Investigation of garden waste heap samples was complicated by high contents of Mucorales interfering with *A. fumigatus* isolation. Consequently, it was only possible to isolate 12 individual *A. fumigatus* isolates, 11 of which harbored TR_34_/L98H (n = 7), TR_46_/Y121F/T289A or TR_46_/Y121F/T289A/S363P/I364V/G448S (n = 3), or F46Y/M172V/E427K (n = 1) *Cyp51A* alterations. The samples from a vegetable composting heap all grew *A. fumigatus* fungi, AR*Af*, and TR_34_/L98H; and 3/5 samples also grew TR_46_/Y121F/T289A or TR_46_/Y121F/T289A/S363P/I364V/G448S. Moreover, many samples grew >200 colonies/plate. Isolation from voriconazole/posaconazole susceptibility plate wells yielded 21 single AR*Af* isolates, of which 81% harbored TR_34_/L98H- and 19% TR_46_/Y121F/T289A-related mechanisms. However, the true number of resistant isolates was probably higher because genotyping suggested mixed genotypes in isolates with a single resistance mechanism.

#### Manure Heaps and Stable Bedding

Of 12 manure heap samples, 8 contained *A. fumigatu*s isolates; the highest isolate numbers were in the 4–5-month-old manure heap at the center 1 (7.7 isolates/sample vs. 3.8 isolates/sample at center 2 with frequent emptying). AR*Af* isolates were found at center 1 (3/5 samples and 25.9% of isolates, all harboring TR_34_/L98H or TR_34_/T-67G/L98H) but not at center 2. Sampling of used stable bedding and the same unused material documented AR*Af* (TR_34_/L98H and TR_46_/Y121F/T289A) in stable bedding but not in unused straw or peat (Table 1).

#### Azole Fungicide

Concentrations in environmental samples were determined for 8 azole fungicides (Appendix Table 8). Levels were generally low and without correlation to AR*Af* detection. Hypothesizing, that a selective fungicide concentration should be at least 1 tenth of the mean MIC against wild-type *A. fumigatus* fungi, we found such concentrations for prothioconazole-desthio in 18 field soil samples (range 9.8–42.9 μg/kg), one of which was AR*Af* positive; for metconazole (38.4–135 μg /kg) in 4 potted plant samples, 3 of which contained AR*Af*; and for difenoconazole (367–717 μg /kg) in 4 field samples, none of which contained AR*Af*. In contrast, AR*Af* was found in 4 potato samples, 1 cactus pot soil, 8 flower bed samples, 5 compost, and 2 wood paint–associated samples with no or very low azole fungicide concentrations (*25*).

### Molecular Characterization of *A. fumigatus* Isolates

Molecular analyses of the 194 resistant and 38 comparator study isolates demonstrated 139 microsatellite genotypes (Table 2). A total of 103 genotypes were found among the resistant isolates and 37 genotypes among susceptible isolates. One genotype was shared among a susceptible and a nonsusceptible isolate (both wild-type *cyp51A*).

**Table 2:** Overview of Cyp51A and Hmg1 genotypes of azole-resistant *Aspergillus fumigatus* isolates, sorted by susceptibility classification, Denmark, 2020–2022

Susceptibility classification and *A. fumigatus* protein alterations	No. genotypes
38 azole-susceptible comparator isolates: 20 Cyp51A wild types	37
6 F46Y/M172V/E427K	
1 M172V	
1 I242V	

10 susceptible isolates, no Cyp51A profile, mixed genotypes	
18 azole-nonsusceptible isolates (9.3% of all nonsusceptible isolates);* 18 Cyp51A wild-types	18
11 Hmg1: Wild type	
1 Hmg1: F262-DEL	
1 Hmg1: W272L (and E105K)	
1 Hmg1: E306K	
3 Hmg1: E105K (outside the sterol-sensing domain)	
1 Hmg1: S541G (outside the sterol-sensing domain)	
22 TR_46_ isolates (11.3%)	13
9 TR_46_/Y121F/T289A	7
10 TR_46_/Y121F/T289A/S363P/I364V/G448S†	5
3 TR_46_**^3^**/Y121F/M172I/T289A/G448S‡	1
154 TR_34_ isolates (79.4%)	72
137 TR_34_/L98H	64
14 have a unique variant in the promotor (T-67G)§	3
3 TR_34_/L98H/S297T/F495I¶	1

*Isolates that were resistant for >1 triazoles on >1 MIC determination. †From flower bed in a private garden in 2021, flower pot soil in 2021, green waste and garden waste compost heaps in 2022, and from field soil in 2022. ‡From flower bed in another private garden in 2021. §From flower bed in a public park 2021, horse manure heap in 2022. **¶**From flower pot soil in 2021.

**Figure 1:**
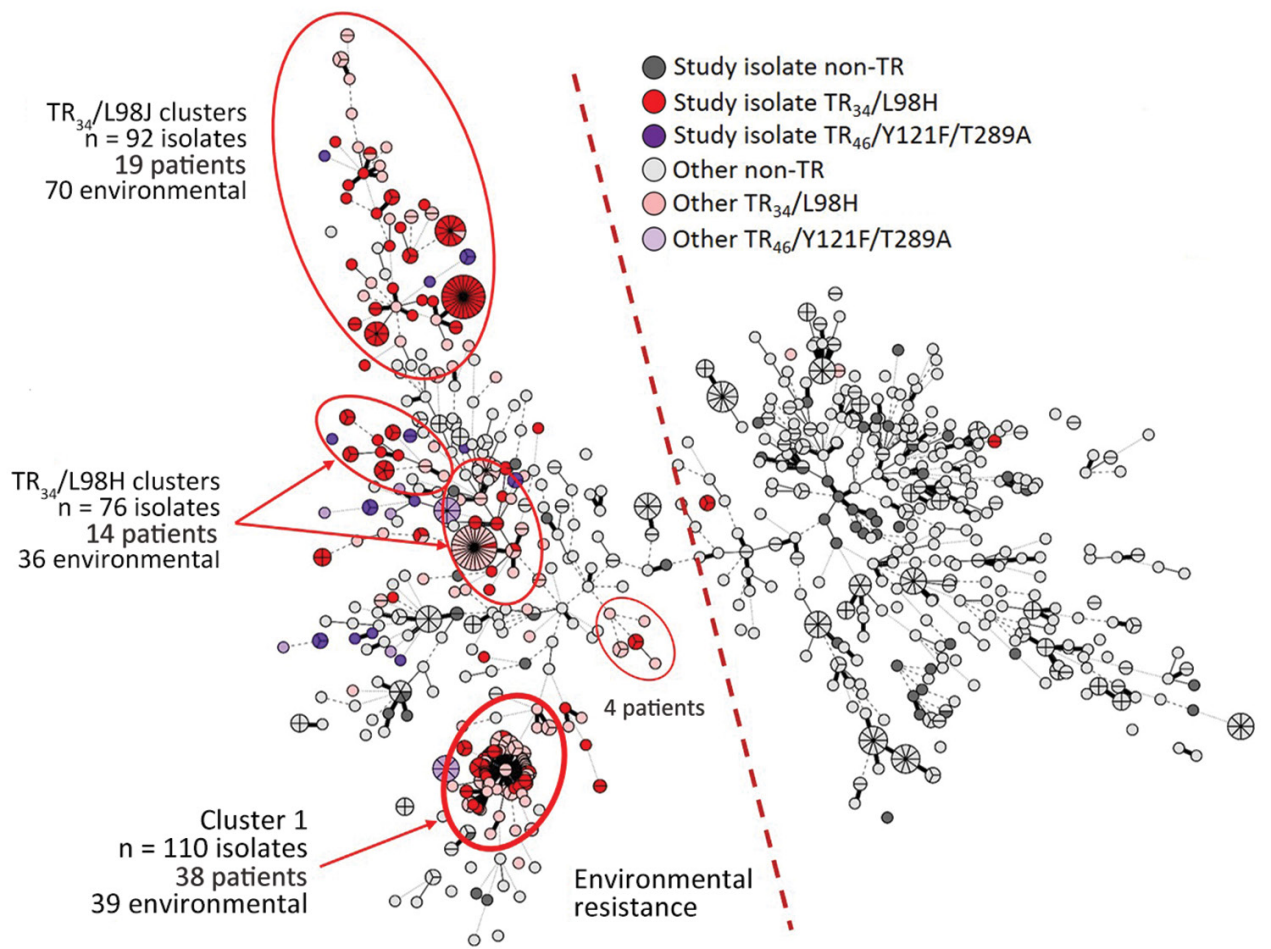
Minimum spanning tree of 232 *Aspergillus fumigatus* genotyped study isolates including 741 Denmark background isolates (627 isolates from 326 patients and 114 isolates from the environment) for study of environmental hot spots and resistance-related application practices for azole-resistant *A. fumigatus*, Denmark, 2020–2022. Colors emphasize isolates with environmental azole resistance mechanisms, TR_34_/L98H (red) or TR_46_/Y121F/T289A (purple). With a few exceptions, all TR_34_/L98H and TR_46_/Y121F/T289A reside on the left side of the tree. Moreover, several TR_34_/L98H clusters include patient and environmental isolates, of which cluster 1 displays almost identical genotypes.

**Figure 2:**
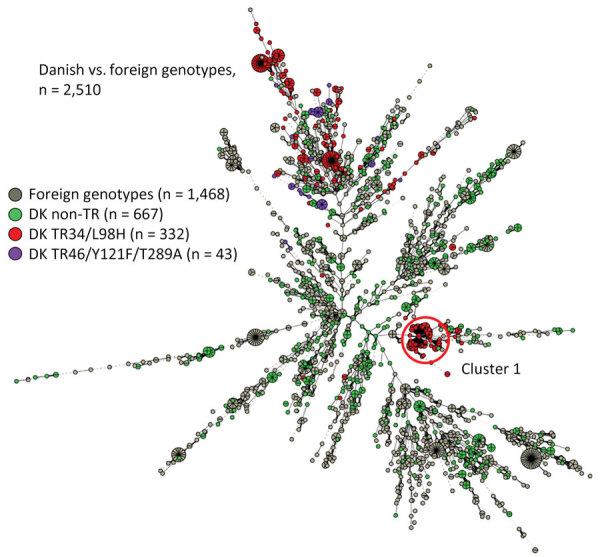
Minimum spanning tree of 1,042 *Aspergillus fumigatus* genotypes from Denmark (green, red, and purple) compared with 1,468 genotypes from other countries (gray) as part of study of environmental hot spots and resistance-related application practices for azole-resistant *A. fumigatus*, Denmark, 2020–2022. The isolates from other countries were mostly azole-resistant *A. fumigatus* and dominated by TR_34_/L98H (F. Hagen, Westerdijk Fungal Biodiversity Institute, pers. comm., 2024 Apr 28). Numbers of isolates from other countries: the Netherlands, n = 615; Norway, n = 209; Belgium, n = 108; Germany, n = 100; Spain, n = 219; United States, n = 102; other, n = 115).

The TR_34_/L98H study isolates included 1 main cluster-1 of 34 TR_34_/L98H identical or closely related isolates from 14 different sampling sites (Figure 1). Other isolate clusters primarily reflect multiple isolates cultured from the same sites. Among the 22 TR_46_-AR*Af* study isolates, 13 unique genotypes were found. One final comparison introduced 1,468 worldwide genotypes from 16 countries (Figure 2). The genotypes from Denmark were widely distributed, corresponding to the worldwide diversity of genotypes. Most AR*Af* isolates were gathered in the top, except for all cluster-1 AR*Af* isolates, which were placed to the right.

### Microcosmos Experiments for AR*Af* Selection

**Figure 3:**
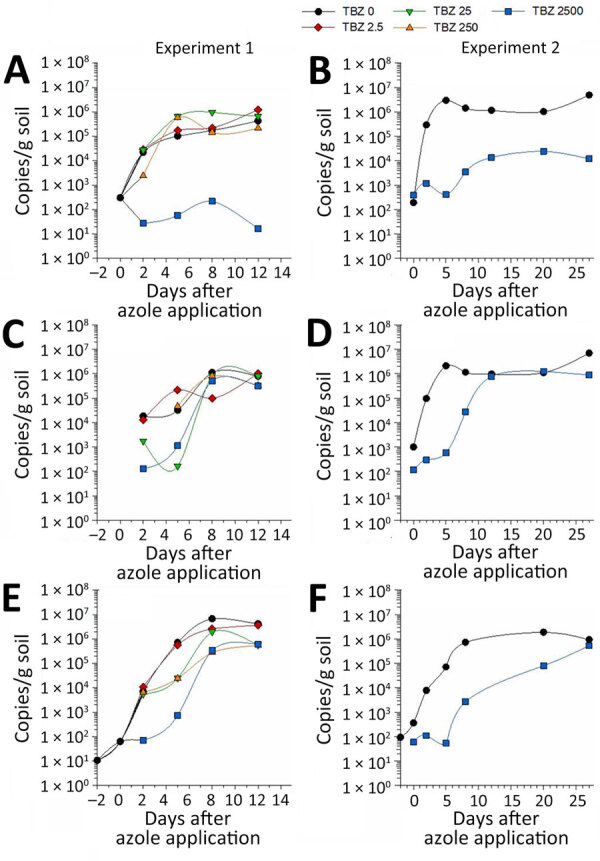
Selective pressure of TBZ on *Aspergillus fumigatus* wild type (A, B), TR_34_/L98H (TR34) (C, D), and TR_46_/Y121F/T289A (TR46) (E, F) in sandy soil (n = 1) in 2 independent microcosmos experiments as part of a study of environmental hot spots and resistance-related application practices for azole-resistant *A. fumigatus*, Denmark, 2020–2022. In experiment 1, in which 4 different concentrations of tebuconazole were used to spike the microcosmos: 2.5 mg/L (red line), 25 mg/L (green line), 250 mg/L (orange line), and 2,500 mg/L (blue line), and growth was followed over 14 days. Growth was quantified by measuring copies of the *cyp51A* promoter region by quantitative PCR. Growth was quantified from day −2 after azole application (the day of inoculation) for the untreated samples and from day 2 after azole application for the samples treated with TBZ. In experiment 2, the effect of the 2,500 mg/L treatment was repeated, and growth was followed over 27 days. Growth was quantified from the day of inoculation (2 days before TBZ application) by measuring copies of the *cyp51A* promoter region by droplet digital PCR. The experiment with TR_46_/Y121F/T289A was in a different microcosmos trial than for wild type and TR_34_/L98H but followed the same protocol. TBZ, tebuconazole.

Wild-type *A. fumigatus*, TR_34_/L98H, and TR_46_/Y121F/T289A failed to grow at 10°C but grew equally well to a maximum of 10^6^–10^7^ CFU/g in heat-sterilized organic rich and sandy soil at 15°C and 20°C (Appendix Figures 1, 2). Sustained complete inhibition was found for wild-type *A. fumigatus* fungi but not AR*Af* at the highest tebuconazole concentration (≈49 mg/kg wet weight) (Figure 3). Prothioconazole conferred initial growth inhibition for all strains, but growth appeared on day 5 or 8 after application and reached the levels of the untreated controls for the TR_34_/L98H and TR_46_/Y121F/T289A strains but not for the wild type (Appendix Figure 3). In contrast, treatment with mefentrifluconazole inhibited growth during the entire microcosmos experiment except for a single replicate with TR_34_/L98H day 27 (Appendix Figure 3).

### Field Experiment for AR*Af* Selection

Of the prespraying and postspraying samples obtained from untreated and azole-treated field sites (Table 3), all samples contained *A. fumigatus* isolates (n = 360, mean 9 isolates/sample), but for all fields, numbers declined 2-fold over time. Ten (2.8%) AR*Af* isolates were found, 4 in unsprayed soil (4/167 = 2.4%) and 6 in treated soil (6/193 = 3.1%, p = 0.757). Seven harbored TR_34_/L98H, 4 found in untreated soil and 3 found after the first prothioconazole spraying in Flakkebjerg. One harbored TR_46_/Y121F/T289A and was found after the third tebuconazole spraying. Two isolates harbored Hmg1 alterations, of which the W272L alteration is situated within the sterol-sensing domain. Those 2 isolates were found after treatment with mefentrifluconazole and prothioconazole. Overall, the resistance percentage increased numerically (p>0.05) from 2.5% before spraying to 6.3% in the first postspraying samples and then declined by 2.5%, 1.9%, and 0 in the remaining postspraying samples. The percentages of AR*Af* harboring tandem repeat mechanisms followed the same pattern.

**Table 3:** Azole-resistant *A. fumigatus* isolates found among *A. fumigatus* isolates obtained at each sampling date in 2 winter wheat fields testing different azole-fungicides applied 2 times for control of leaf diseases, Denmark, 2020–2022*

Sample sites and treatments and doses	Sampling related to spraying

Before first spray	Before second spray	Week 3 after second spray	Week 6 after second spray	Week 10 after second spray
Flakkebjerg					
Untreated control field	0/8	0/10	0/10†	0/3†	0/2†
Prothioconazole 2 × 0.4 L/ha	0/29	**3**/12	0/16†	0/5†	0/6†
Tebuconazole 2 × 0.5 L/ha	**1**/16	0/11	0/13†	**1**/9†	0/9†
Mefentrifluconazole 2 × 0.75/ha	**1**/11	**1**/13	0/14†	0/5†	0/4†
Fredericia					
Untreated control field	0/12	0/6	**1**/6	0/9	0/3
Prothioconazole 2 × 0.4 L/ha	0/15	0/2	**1**/7	0/4	0/6
Tebuconazole 2 × 0.5 L/ha	**1**/13	0/7	0/3	0/9	0/12
Mefentrifluconazole 2× 0.75 L/ha	0/14	0/2	0/11	0/8	0/5
AR*Af* isolates/*A. fumigatus* (per date)	3/118	4/63	2/80	1/52	0/47
Isolates w. TR_34_ or TR_46_, no. (%)	3 TR_34_ (2.5)	3 TR_34_ (4.8)	1 TR_34_(1.3)	1 TR_46_ (1.9)	0
Isolates w. Hmg1 alterations		1 Hmg1 E105K (1.6)	1 Hmg1 E105K/ W272L (1.3)		

*Green indicates findings from untreated sites (before spraying or control sites); gray indicates findings from sprayed sites. Sampling dates in 2020 were May 15, May 30, Jun 20, Jul 11, and Aug 12 for Flakkebjerg and May 12, May 30, Jun 17, Jul 12, and Aug 8 for Fredericia. Boldface indicates resistant isolates. *Af*, *Aspergillus fumigatus*; AR*Af*, azole-resistant *A. fumigatus.*†Sites that received an additional tebuconazole third spray on June 19, 2022, to stop a rust disease outbreak.

## Discussion

Our study demonstrated that AR*Af* is extensively distributed in the environment in Denmark. AR*Af* was found in 20% of 366 samples, and 4.2% of 4,538 investigated isolates were azole resistant, dominated by TR_34_/L98H-related and, to a lesser extent, TR_46_/Y121F/T289A-related mechanisms. Although the study was not designed to capture longitudinal changes, 3 observations suggest that AR*Af* is increasing in Denmark. First, although AR*Af* percentages were relatively low (0.3%–2.8%) among *A. fumigatus* isolates in agricultural soil samples from Denmark, they were higher than in studies conducted in 2010 and 2013, where no AR*Af* was found among 113 *A. fumigatus* isolates from flower beds, potted plants, and conventional and organic fields (*7*). Second, the AR*Af* proportion was higher in field air in 2021 than in 2020 (p = 0.0202) and higher in field soil in 2022 than in 2020 (p = 0.0127). Third, TR_46_/Y121F/T289A was found in multiple settings during 2021–2022 but not in 2020, despite a comparable number of samples. Those findings coincide with the first of several isolations of TR_46_/Y121F/T289A from patients in Denmark in 2021 (M.C. Arendrup, unpub. data) since the initial finding of this genotype in a single patient in 2014 (*7*). Genotyping identified a nationwide cluster of TR_34_/L98H with wide geographic distribution across Denmark, including clinical and environmental isolates. That particular clone has remained dominant among azole-resistant clinical isolates from Denmark since 2018. Whether that trait of augmented mutation rate is a virulence factor and responsible for the relatively high prevalence among Denmark AR*Af* warrants further investigation, but it aligns with the observed increasing incidence. It is also of interest that that cluster is located quite distant from most other tandem repeat isolates, possibly indicating that that clone has appeared through sexual recombination of unrelated strains.

The AR*Af* isolates were more closely related than the *A. fumigatus*–susceptible isolates. That finding suggests more recent ancestors and that the increasing environmental resistance rates are driven mainly by factors favoring propagation of TR_34_/L98H and TR_46_/Y121F/T289A genotypes already present over the susceptible population rather than induction of resistance in susceptible isolates from outside.

Besides characterizing the prevalence of AR*Af* and relevant hot spots in Denmark, it was our intention to investigate potential links between the presence of azoles in the samples and AR*Af*. Azoles were found at low concentrations in most soil samples, indicating persistence of azoles in the soils and a measurable carryover concentration from season to season (data not shown). We saw no association between AR*Af* findings and azole concentration in any specific sample or across sample types, nor did we verify increasing resistance after azole spraying in wheat field trials, potentially because *A. fumigatus* growth was absent. In contrast, our microcosmos experiments suggested that azole fungicides may favor AR*Af* growth over wild-type *A. fumigatus* in soil. Few studies have been able to confirm a link between specific azole use and resistance in *A. fumigatus* (*3*). A study in China indicated a link between use of azoles in paddy rice and resistance development, whereas a recent study in Switzerland found that azole resistance was neither associated with any specific agricultural practice nor with the presence of azole fungicides (*26*,*27*). The concentration of azoles in an environmental sample is a snapshot, which fails to provide information about previous exposures, potentially relevant for resistance selection. Other factors may influence selection and presence of AR*Af* at the time of sampling (e.g., soil type, temperature, humidity, competition from the indigenous microbial community, azole application concentration and subsequent kinetics of free and soil bound fractions, liquid manure application, and amount of organic matter). Those factors complicate identification of safe and unsafe procedures, particularly as the annual increase in AR*Af* appears to be well below 1% in Denmark, suggesting a slow and potentially fluctuating increase that is difficult to capture in light of the heterogeneity of environmental samples.

We confirmed that the hot spots for AR*Af* are compost, flower beds, and flower production; but we also found AR*Af* in stables and horse manure heaps (*1*,*3*,*28*). Azoles are not used in parks, gardens, or stables. However, planting azole-treated bulbs and using compost soil based on azole-containing plant material can turn flower beds and garden waste heaps into hot spots (*29*). Of note, the tulip cultivars found in the private gardens were old cultivars and the azole contents were very low, suggesting that the AR*Af* found could reflect the general background AR*Af* population combined with good growth conditions for *A. fumigatus* rather than a direct link to azole-treated bulb planting. In addition, our study findings suggest that it is plausible that use of azole-containing conventional straw for stable bedding similarly can turn stable bedding and manure heaps into hot spots and thus reflect collateral damage associated with azole use elsewhere.

One limitation of our study is that the sensitivity for AR*Af* detection in a given sample and sample type will vary because of the variable number of *A. fumigatus* in the environmental samples. Consequently, we cannot exclude that AR*Af* may be found in negative samples, in which *A. fumigatus* numbers were low. Yet it is plausible that such settings, because of the overall lower *A. fumigatus* prevalence, may contribute less to resistance selection and human exposure. Another limitation is that we did not have funding for whole-genome sequencing. However, microsatellite typing has been widely used and has a high discriminative power, enabling us to compare with already published data.

In conclusion, our study and the available literature strongly suggest that the dual use of azoles in clinical medicine and for crop and material protection has introduced azole resistance in *A. fumigatus*, which challenges patient management. Isolates harboring environmental resistance mechanisms were found in every setting explored and expand the numbers, genotypes, and target gene variants found in earlier studies. Because of a lack of fitness cost (*30*), the AR*Af* variants will remain even if use of azoles active against *A. fumigatus* is terminated. It seems advisable to avoid future dual use of agents used in human medicine, such as drug candidates olorofim and fosmanogepix, which are threatened by new compounds developed for plant protection (*31*). Prioritizing the use of *A. fumigatus* active azole fungicides might potentially slow the rise in rates of resistance. 

Appendix Additional information for study of environmental hot spots and resistance-associated application practices for azole-resistant *Aspergillus fumigatus*, Denmark, 2020–2022.
