# Supplementary material for: Environmental Hot Spots and Resistance-Associated Application Practices for Azole-Resistant Aspergillus fumigatus, Denmark, 2020–2023
Source: Emerg Infect Dis. 2024 Aug;30(8):1531–41. doi: 10.3201/eid3008.240096 (PMC11286046; doi:10.3201/eid3008.240096)
Supplement: Appendix — Additional information for study of environmental hot spots and resistance-associated application practices for azole-resistant Aspergillus fumigatus, Denmark, 2020–2022. [file 24-0096-Techapp-s1.pdf]

Article DOI: https://doi.org/10.3201/eid3008.240096

EID cannot ensure accessibility for supplementary materials supplied by authors. Readers who have difficulty accessing supplementary content should contact the authors for assistance.

Environmental Hot Spots and Resistance-Associated Application Practices for Azole-Resistant *Aspergillus fumigatus*, Denmark, 2020–2022

Appendix

Appendix Table 1. Overview of sample types, dates and characteristics. Unless otherwise specified, samples were split and analysed both by SSI (AR*Af*) and GEUS (azoles).

Sample type	Date	*N.* of samples	Comments
Fields			
Air	August 2020	51 divided between two localities	Air samples were collected using a Sartorius air sampler, with subsequent application of exposed gelatine filters directly on agar culture plates. Sampling was done both before, during and after harvesting at two localities (Flakkebjerg (Zealand), Dannemare (Lolland). Fields were treated with standard fungicides including prothioconazole and tebuconazole. No soil samples were taken from the fields where air samples were collected. Air samples were not applicable for azole concentration determination.
Soil	August 13-24th 2020	2 sites x 10 samples =20	Fields from the pesticide leaching assessment programme (www.plap.dk; Fårdrup (Zealand), Silstrup (Jutland). Samples were taken after harvest of cereal crops from areas, which have a known history of azole treatments as detailed in Table 5
Soil	September 22nd 2020	3 sites x 4 samples from untreated 3 sites x 4 samples from azole treated =24 samples	Potatoes fields (Flakkebjerg (Zealand), Dronninglund (Jutland), Herning, Jutland)) (Table 6). Samples were taken at sites, where 4-5 treatments with azoles (difenoconazole and or prothioconazole) were applied for control of Alternaria on potatoes. Both untreated and treated plots were sampled.
Air	August 2021	38 divided between two localities	Air samples were collected using a Sartorius air sampler, with subsequent application of exposed gelatine filtres directly on agar culture plates. Sampling was done both before, during and after harvesting at two localities (Flakkebjerg, Dannemare). Fields were treated with standard fungicides including prothioconazole and tebuconazole. No soil samples were taken from the fields where air samples were collected. Air samples were not applicable for azole concentration determination.
Soil	Summer 2022	5 sampling times from 4 treatments x 2 locations = 40 samples	Samples were taken from two wheat trial sites (Fredericia (Jutland), Flakkebjerg (Zealand)) where two treatments with different azoles (prothioconazole, tebuconazole, mefentrifluconazole) were been applied twice (Table 7). Both untreated and treated plots were sampled, before spraying, before the 2nd spray and after 3, 6 and 10 weeks after the 2nd spray. Each sample included soil from 4 replicates. The Flakkebjerg site was further treated with 0,25 Folicur 250 EC (tebuconazole) to reduce a risk of yellow rust, which unintededly developed in the wheat trial, which had focus regarding control of Septoria tritici blotch.
Flower beds & Flower producers			
Soils	April 24th and May 17th 2021	4 parks x 3 samples (2 x T, 1 x K, 1 x F) 2 gardens x 3 samples	Samples were taken at three flower beds per park/garden. Three public parks North Zealand and near Copenhagen) and two private gardens (Flakkebjerg and Copenhagen) were sampled. Samples were all taken in connection with spring flowers (tulips and other bulb plants).

Sample type	Date	*N*. of samples	Comments
		=18 samples	None of the parks nor gardens had any history of azole fungicides application in the recent history.
Soil	Nov 9th 2020	2 x 10 pots with poinsettia<br>1 x 10 cactus<br>1 x 10 campanula<br>= 40 samples	Samples were taken from the greenhouses, growing flower pots (Table 8). Sampling was done just before the plants were ready to be sold. Potted plants had all been treated with either metconazole or paclobutrazole used as a plant growth regulator. Pot topsoil (top 5 cm) from 4 different cultures from Fyn were analysed.
Soil	2021	10 samples of poinsettia	One of the flower growers from 2020 was revisited and poinsettia was re-sampled. Top soil from flower pots was analysed. Air samples were also taken from the same grower's greenhouses.
Air	2021	10 samples	Air was sampled from greenhouses with production of poinsettia using Sartorius air sampler with gelatine filter application on agar plates.
Vegetables			
Potato peels	November 23rd 2020	10 sources x 4 samples each =40 samples for	6 potato-samples from 6 supermarkets (Vildmose with remains of soil), 2 from a farm shop and 2 from potatoes harvested at Flakkebjerg. Potato peel from potatoes were sampled (no pre-washing) and cut into small pieces.
Compost and manure			
Compost soil (Garden waste)	May 2022	5 samples	Samples at different depths were collected from the final "ready to use" compost soil at a regional recycle unit in the Greater Copenhagen area.
Composting heap (Garden waste)	November 2022	10 samples	Samples were taken from a regional compost unit (Affald plus Næstved Zealand), which was based on plant material from private gardens. Five samples were taken from the surface and five from approx. 10-20 cm inside the heap. Each sample was a mix of 5 subsamples.
Composting heap (vegetable waste)	November 2022	5 samples	Samples taken from a vegetable grower's composting heap. The heap contained residual plant material from the production of different vegetables, which is removed before being sold. Each sample was picked approximately 10 cm inside the heap.
Horse dung etc.	Feb/March 2022	20 samples	Dung containing manure heap (long-term) (n=8) (North Zealand). Dung containing box-bedding in stables using bedding of straw (wheat & barley) or peat (n=7) and unused straw and peat were also obtained (n=6).
	Nov 2022	5 samples	"Short-term" manure heap in a container at a horseback riding school in Copenhagen.
Paint related			
Soil	September 2021	14 samples	Samples were obtained in close proximity to painted/treated wooden allotment houses, fences and terraces. Paint history and sample description were obtained via a questionnaire completed by the allotment house owners (see below under results); additionally, the wooden surfaces were flushed with water and the run-off water was analysed for azoles qualitatively.

Appendix Table 2. Azole compound specific mass spectrometric parameters, limits of detection (LOD)[a] and quantification (LOQ)[a]

[illegible]	[illegible]	Quantifier (Collision energy)	Qualifier (Collision energy)	Surrogate standard	[illegible]	LOQ [µg/kg]
[illegible]	[illegible]	342 → 69 (CE 19V)	342 → 159 (CE 25V)	PPZ-D5	[illegible]	0.8
[illegible]	[illegible]	308 → 70 (CE 18V)	310 → 70 (CE 32V)	TBZ-D9	[illegible]	0.8
[illegible]	[illegible]	330 → 121 (CE 19V)	330 → 70 (CE 18V)	TBZ-D9	[illegible]	1.1

[illegible]	[illegible]	Quantifier (Collision energy)	Qualifier (Collision energy)	Surroga te standar d	[illegible]	LOQ [µg/kg]
[illegible]	[illegible]	320 → 70 (CE 20V)	320 → 125 (CE 30V)	PPZ-D5	[illegible]	1.1
[illegible]	[illegible]	406 → 251 (CE 24V)	406 → 118 (CE 54V)	DFZ-D6	[illegible]	1.0
[illegible]	[illegible]	312 → 70 (CE 22V)	312 → 125 (CE 34V)	PPZ-D5	[illegible]	0.9

[illegible]	[illegible]	Quantifier (Collision energy)	Qualifier (Collision energy)	Surrogate standard	[illegible]	LOQ [µg/kg]
[illegible]	[illegible]	294 → 70 (CE 18V)	294 → 125 (CE 45V)	TBZ-D9	[illegible]	0.5
[illegible]	[illegible]	398 → 70 (CE 22V)	398 → 182 (CE 28V)	TBZ-D9	[illegible]	0.8

[illegible]	[illegible]	Quantifier (Collision energy)	Qualifier (Collision energy)	Surrogate standard	[illegible]	LOQ [µg/kg]
[illegible]	[illegible]	312 → 70 (CE 22V)	312 → 125 (CE 34)			

[illegible]	[illegible]	Quantifier (Collision energy)	Qualifier (Collision energy)	Surrogate standard	[illegible]	LOQ [µg/kg]
[illegible]	[illegible]	347 → 74 (CE 19V)	347 → 159 (CE 25V)			

[illegible]	[illegible]	Quantifier (Collision energy)	Qualifier (Collision energy)	Surrogate standard	[illegible]	LOQ [µg/kg]
[illegible]	[illegible]	412 → 251 (CE 27V)	312 → 343 (CE 18V)			

[illegible]	[illegible]	Quantifier (Collision energy)	Qualifier (Collision energy)	Surroga te standar d	[illegible]	LOQ [µg/kg]

[a] LOD and LOQ were determined as 3-times (LOD) and 10-times (LOQ) standard deviation of a control sample spiked at a low concentration. Empty cells indicate no surrogate standard available.

Appendix Table 3. PCR and sequencing primers used for *cyp51A* sequencing of azole-resistant *A. fumigatus* isolates

Primer ID	5′ – 3′ sequence	Purpose	Reference
0F	TCATATGTTGCTCAGCGG	PCR	(*1*)
1F	GTGCGTAGCAAGGGAGAAGGA	Sequencing	*In house*
1R	CATTGAGCAAGATTGCCG	Sequencing	(*2*)
2F	CGGCAATCTTGCTCAATG	Sequencing	(*2*)
2R	GGTGAATCGCGCAGATAGT	Sequencing	(*2*)
3F	ACTATCTGCGCGATTCACC	Sequencing	(*2*)
3R	GTCAAGATCCTTGTACTGGAGC	Sequencing	(*2*)
4F	GCTCCAGTACAAGGATCTTGAC	Sequencing	(*2*)
4R	CCTATTCCGATCACACCAAA	PCR & Sequencing	(*2*)

Appendix Table 4. PCR and sequencing primers used for *hmg1* sequencing of azole-resistant *A. fumigatus* isolates

Primer ID	5′ – 3′ sequence	Purpose	Reference
HMG-S1	CGAGTCGAGAGAATTTGAGACG	PCR 1 & Sequencing	This study
HMG-S2	CGTCAACCATCTTGCCTTAACA	Sequencing	This study
HMG-S3	GCCTTCCATTCCTTGTTGTCA	PCR 2 & Sequencing	This study
HMG-S4	TCGTGTCTCTAATGTGTTTGCG	Sequencing	This study
HMG-S5	CCGTGTGGATGCGTTCACTA	Sequencing	This study
HMG-S6	CACCGCTTTCAGAAATTGTGG	Sequencing	This study
HMG-S7	TTGTCGCAACCCACCCTG	Sequencing	This study
HMG-S8	GGTCAGAGCAGCAATGATCCA	PCR 1 & Sequencing	This study
HMG-S9	GGCAAACCGACTGGTAGAGTTA	Sequencing	This study
HMG-S10	CTCGGCTACATTCCTCTCTTGG	PCR 2 & Sequencing	This study

Appendix Table 5. Primers and repeat units for the STR*Af* microsatellite genotyping assay for *A. fumigatus* (*3*)

Multiplex	Marker ID	Forward primer (5′ - 3′)	Reverse primer (5'-3')	Repeat unit
M2	STR*Af* 2A	FAM-AAGGGTTATGGCCATTAGGG	GACCTCCAGGCAAAATGAGA	GA/TC
	STR*Af* 2B	HEX-TATTGGATCTGCTCCCAAGC	GAGATCATGCCCAAGGATGT	AG/CT
	STR*Af* 2C	NED-TCGGAGTAGTTGCAGGAAGG	AACGCGTCCTAGAATGTTGC	CA/TG
M3	STR*Af* 3A	FAM-GCTTCGTAGAGCGGAATCAC	GTACCGCTGCAAAGGACAGT	TCT/AGA
	STR*Af* 3B	HEX-CAACTTGGTGTCAGCGAAGA	GAGGTACCACAACACAGCACA	AAG/CTT
	STR*Af* 3C	NED-GGTTACATGGCTTGGAGCAT	GTACACAAAGGGTGGGATGG	TAG/CTA
M4	STR*Af* 4A	FAM-TTGTTGGCCGCTTTTACTTC	GACCCAGCGCCTATAAATCA	TTCT/AGAA
	STR*Af* 4B	HEX-CGTAGTGACCTGAGCCTTCA	GGAAGGCTGTACCGTCAATCT	CTAT/ATAG
	STR*Af* 4C	NED-CATATTGGGAAACCCACTCG	ACCAACCCATCCAATTCGTAA	ATGT/ACAT

Appendix Table 6. Azole concentrations in microcosmos.

Spike solution (mg/L)	Estimated wet weight concentration in 5.1 g sample (mg/kg ww)	Estimated dry weight concentration in 4 g dry sample (mg/kg dw)
2.5	0.049	0.0625
25	0.49	0.625
250	4.9	6.25
2,500	49	62.5

Appendix Table 7. Primers and probes for *A. fumigatus* quantification PCR assays[a]

Oligo name	5′ - 3′ sequence	Reference
Assay (1) *A. fumigatus* specific detection targeting ITS.		
AF-ITS-F2	CGAAGACCCCAACATGAACG	In house
AF-ITS-R2	ATTCTGCAATTCACATTAC	In house
AF-ITS-P2	VIC-CTGTTCTGAAAGTATGCAGTCTG	In house
Assay (2) *A. fumigatus* and specifically TR_{34}/L98H or TR_{46}/Y121F/T289A detection targeting *cyp51A*		
AF-TR-F3	GTGAATAATCGCAGCACCAC	In house
AF-TR-R6	CCTACCAATATAGGTTCATAGGTAAG	In house
AF-CYP-P3	FAM-AGTTAGGGTGTATGGTATGCTGGAAC-BHQ1	In house
AF-TR34-P3	VIC-GCTGAGCCGAATGAATCACGC- BHQ1	In house
AFCYP-TR46-P9	VIC-CGAATGAAAGTTGTCTAGAATCACGC*-BHQ1	In house

[a] PCR conditions for both assays were as follows; 1x Quanta ToughMix® (dNTPs, polymerase, buffer and "low ROX", QuantaBio distributed by VWR, Søborg, Denmark), 0.5 µM forward and reverse primer, 0.4 µM probe for assay (1) and 0.33 µM probe for assay (2), 0.8 mM and 3.5 mM additional MgCl2 for assay (1) and (2), respectively, 5 µL DNA and molecular biology grade Gibco water (Thermo Fisher Scientific, Roskilde, Denmark) in 30 µL reaction volumes. All PCR reactions were run on the Quantstudio 5, thermocycler (Thermo Fisher Scientific, Roskilde, Denmark) with 5 min denaturation at 95 oC followed by 45 cycles of denaturation (20 s at 95 oC) and annealing (1 min at 60 oC) with data acquisition at this step and ramp rates of 1.6 oC/s. PCR data was analysed with the Quantstudio Design & Analysis Desktop software v1.5.2 (Applied Biosystems by Thermo Fisher Scientific, Denmark). Sensitivity was evaluated by 10-fold dilution series of normalised DNA concentrations and spore suspensions, extracted on Nuclisens easyMag (biomérieux, Denmark), using 1 mL input volume of supernatant from bead-beated spore suspensions and 60 µL elution volume. The limit of detection (LOD) was estimated to 50 fg DNA (<20 copies) and 50-200 CFU/mL, respectively with no differences between the three probes (A. fumigatus *cyp51A*, TR34 and TR46) (data not shown).

*Previously, this probe was labelled with ABY fluorescence dye to enable triplexing with the CYP-P3 and the TR34-P3 probes and to enable detection of all three in a single PCR reaction. The results, however, indicated cross-reaction and undesired interference of fluorescence, and thus for the majority of the samples the TR46-probe was either run as duplex with the CYP-P3 FAM probe or alone.

A. fumigatus quantification in microcosmos experiments:

For the first experiments, the target was a multicopy internal transcribed spacer (ITS, Assay (1)). Ct value to CFU/g soil conversion was based on a standard curve with known CFU and Ct values (log(X)=(Y-34,137)/-3,5178), where "X" = CFU/PCR reaction and "Y" = Ct value (data not shown).

For subsequent experiments primers and probes targeting the cyp51A promoter were used (Assay (2)) able to distinguish TR34(/L98H) and TR46(/Y121F/T289A). Standard curves were calculated as copies (=CFU) based on weighed DNA content of the controls and converted using a conventional conversion formula: Number of copies = (ng (weight of DNA) x [Avogadro's number])/(length (genome) x [1 x 109] x 650 g/mol).

Droplet PCR:

Each PCR sample contained 1x ddPCR Supermix for probes (no dUTP) (Biorad), 1 µM AF-TR-F3 forward primer, 1 µM AF-TR-R6 reverse primer, 0.5 µM AF-CYP-P3 probe, 2.5 µL or 5 µL DNA extract and nuclease-free water (Qiagen) in a total volume of 20 µL. Primer and probe sequences are listed in [REMOVED REF FIELD]Appendix Table6. DG8 cartridges (Biorad) were loaded with 20 µL PCR reaction and 70 µL droplet generation oil for probes (Biorad) and droplets were generated in a QX200 droplet generator (Biorad). The final cycling conditions were: 95°C for 10 minutes, 45 cycles of 94°C for 45 seconds and 60°C for 90 seconds with a ramp rate of 2°C/s, followed by 10 minutes at 98°C. ddPCR reactions incubated at 12°C overnight before QX200 droplet reader quantification (Biorad).

Appendix Table 8. Fungicide azole concentration (µg/kg dry weight) determinations in environmental samples

		Concentration [µg/kg dw]							
Sampling site	AR*Af* positive	TBZ	PPZ	MTZ	EPZ	DFZ	PTZ-S	PBZ	MFZ
Field 1	No	0.6	3.4	< 0,3	5.3	2.0	9.9	n.a.	n.a.
Field 1	Yes	1.0	2.1	< 0,3	5.2	2.6	19.8	n.a.	n.a.
Field 1	No	0.9	2.3	< 0,3	5.5	4.9	9.8	n.a.	n.a.
Field 1	No	0.9	5.0	< 0,3	5.2	4.1	10.4	n.a.	n.a.
Field 1	No	0.7	1.2	< 0,3	5.3	24.1	17.6	n.a.	n.a.
Field 1	No	0.7	1.1	< 0,3	5.0	5.2	12.6	n.a.	n.a.
Field 1	No	0.6	1.0	< 0,3	4.2	9.2	10.2	n.a.	n.a.
Field 1	No	0.5	1.4	< 0,3	5.8	1.6	13.8	n.a.	n.a.
Field 1	No	0.6	1.5	< 0,3	5.1	9.1	14.8	n.a.	n.a.
Field 1	No	0.6	2.3	< 0,3	5.2	8.7	11.0	n.a.	n.a.
Field 2	No	2.3	1.8	< 0,3	1.6	< 0,3	0.5	n.a.	n.a.
Field 2	No	2.1	1.6	< 0,3	1.6	< 0,3	0.5	n.a.	n.a.
Field 2	No	2.2	1.6	< 0,3	1.9	< 0,3	0.3	n.a.	n.a.
Field 2	No	2.3	1.7	< 0,3	1.3	< 0,3	0.3	n.a.	n.a.
Field 2	No	2.5	1.5	< 0,3	1.5	< 0,3	0.4	n.a.	n.a.
Field 2	No	2.2	1.5	< 0,3	1.4	< 0,3	0.5	n.a.	n.a.
Field 2	No	2.2	1.1	< 0,3	1.5	< 0,3	0.4	n.a.	n.a.
Field 2	No	2.1	0.7	< 0,3	2.2	< 0,3	0.8	n.a.	n.a.
Field 2	No	2.9	1.5	< 0,3	1.4	< 0,3	0.4	n.a.	n.a.
Field 2	No	2.3	1.2	< 0,3	1.9	< 0,3	0.6	n.a.	n.a.
Field 3 - segment 1	No	0.4	0.3	< 0,3	2.4	0.4	0.4	n.a.	n.a.
Field 3 - segment 1	No	1.2	0.4	< 0,3	1.7	0.4	1.5	n.a.	n.a.
Field 3 - segment 1	No	0.3	0.4	< 0,3	2.0	2.7	0.5	n.a.	n.a.
Field 3 - segment 1	No	< 0.3	0.3	< 0,3	1.6	< 0,3	0.4	n.a.	n.a.
Field 3 - segment 2	No	< 0.3	0.3	< 0,3	1.4	283	0.5	n.a.	n.a.
Field 3 - segment 2	No	0.5	0.3	< 0,3	1.8	717	1.2	n.a.	n.a.
Field 3 - segment 2	No	< 0.3	0.4	< 0,3	2.4	367	0.6	n.a.	n.a.
Field 3 - segment 2	No	< 0.3	0.2	< 0,3	1.7	487	0.4	n.a.	n.a.
Field 4 - segment 1	No	1.0	0.3	< 0,3	11.3	36.4	1.1	n.a.	n.a.
Field 4 - segment 1	No	0.9	0.4	< 0,3	10.0	54.5	1.3	n.a.	n.a.
Field 4 - segment 1	No	1.1	0.5	< 0,3	8.1	116	1.5	n.a.	n.a.
Field 4 - segment 1	No	1.3	0.6	< 0,3	12.8	130	2.3	n.a.	n.a.
Field 4 - segment 2	No	0.8	0.4	< 0,3	10.5	215	24.0	n.a.	n.a.
Field 4 - segment 2	No	1.4	0.4	< 0,3	10.8	391	39.6	n.a.	n.a.
Field 4 - segment 2	No	1.5	0.5	< 0,3	12.1	222	42.9	n.a.	n.a.
Field 4 - segment 2	No	1.0	0.4	< 0,3	9.4	133	11.9	n.a.	n.a.
Field 5 - segment 1	No	2.1	0.3	< 0,3	5.3	11.4	1.1	n.a.	n.a.
Field 5 - segment 1	No	1.1	0.4	< 0,3	2.6	4.6	0.5	n.a.	n.a.
Field 5 - segment 1	No	1.8	0.3	< 0,3	2.8	16.5	1.2	n.a.	n.a.
Field 5 - segment 1	No	1.3	< 0.2	< 0,3	2.6	13.6	0.7	n.a.	n.a.
Field 5 - segment 2	No	1.1	0.3	< 0,3	3.6	83.3	10.2	n.a.	n.a.
Field 5 - segment 2	No	1.7	0.3	< 0,3	3.3	91.2	5.4	n.a.	n.a.
Field 5 - segment 2	No	2.4	< 0.2	< 0,3	3.6	83.5	37.7	n.a.	n.a.
Field 5 - segment 2	No	1.3	< 0.2	< 0,3	2.9	62.1	10.9	n.a.	n.a.
Potato 1	No	< 1.0	< 1.0	< 1.4	< 1.3	1.5	< 1.2	< 1.2	n.a.
Potato 2	No	< 1.0	< 1.0	< 1.4	< 1.3	< 1.2	< 1.2	< 1.2	n.a.
Potato 3	No	< 1.0	< 1.0	< 1.4	< 1.3	< 1.2	< 1.2	< 1.2	n.a.
Potato 4	No	< 1.0	< 1.0	< 1.4	< 1.3	3.6	< 1.2	< 1.2	n.a.
Potato 5	No	< 1.0	< 1.0	< 1.4	< 1.3	< 1.2	< 1.2	< 1.2	n.a.
Potato 6	No	< 1.0	< 1.0	< 1.4	< 1.3	< 1.2	< 1.2	< 1.2	n.a.
Potato 7	Yes	2.5	< 1.0	< 1.4	< 1.3	< 1.2	< 1.2	< 1.2	n.a.
Potato 8	Yes	1.5	< 1.0	< 1.4	< 1.3	< 1.2	< 1.2	< 1.2	n.a.
Potato 9	No	< 1.0	< 1.0	< 1.4	< 1.3	39.7	1.3	< 1.2	n.a.
Potato 10	Yes	< 1.0	< 1.0	< 1.4	< 1.3	12.8	4.4	< 1.2	n.a.
Park 1 - bed 1	No	0.8	< 0.2	< 0,3	< 0,3	< 0,3	5.9	< 0.1	n.a.
Park 1 - bed 2	Yes	1.0	< 0.2	< 0,3	< 0,3	2.1	2.6	< 0.1	n.a.
Park 1 - bed 3	No	0.8	< 0.2	< 0,3	< 0,3	< 0,3	0.6	< 0.1	n.a.
Park 2 - bed 1	No	< 0.3	< 0.2	< 0,3	< 0,3	< 0,3	< 0,3	< 0.1	n.a.
Park 2 - bed 2	Yes	< 0.3	< 0.2	< 0,3	< 0,3	< 0,3	< 0,3	< 0.1	n.a.
Park 2 - bed 3	Yes	< 0.3	< 0.2	< 0,3	< 0,3	< 0,3	< 0,3	< 0.1	n.a.
Park 3 - bed 1	Yes	< 0.3	< 0.2	< 0,3	< 0,3	< 0,3	< 0,3	< 0.1	n.a.
Park 3 - bed 2	Yes	0.4	< 0.2	< 0,3	< 0,3	< 0,3	< 0,3	< 0.1	n.a.
Park 3 - bed 3	Yes	< 0.3	< 0.2	< 0,3	< 0,3	< 0,3	< 0,3	< 0.1	n.a.
Park 4	No	1.9	< 0.2	< 0,3	< 0,3	< 0,3	0.5	< 0.1	n.a.
Garden 1	Yes	< 0.3	< 0.2	< 0,3	< 0,3	< 0,3	< 0,3	< 0.1	n.a.
Garden 2	Yes	0.8	< 0.2	< 0,3	< 0,3	< 0,3	< 0,3	< 0.1	n.a.
Campanula 2020	Yes	< 5	< 5	< 5	< 5	< 5	< 5	4.1	n.a.

Sampling site	ARA*f* positive	Concentration [µg/kg dw]							
		TBZ	PPZ	MTZ	EPZ	DFZ	PTZ-S	PBZ	MFZ
Cactus 2020	No	< 5	< 5	135	< 5	74.0	< 5	20.9	n.a.
Poinsettia A 2020	Yes	< 5	22.7	38.4	< 5	< 5	< 5	< 5	n.a.
Poinsettia B 2020	Yes	< 5	< 5	88.0	< 5	< 5	< 5	< 5	n.a.
Poinsettia 2021	Yes	< 5	< 5	117	< 5	< 5	< 5	70.0	< 5
Compost 1	Yes	0.4	0.6	< 0.3	< 0.3	< 0.3	< 0.3	< 0.1	< 0.3
Compost 2	Yes	0.4	0.6	< 0.3	< 0.3	< 0.3	< 0.3	< 0.1	< 0.3
Compost 3	Yes	0.6	1.1	< 0.3	< 0.3	< 0.3	< 0.3	< 0.1	< 0.3
Compost 4	Yes	0.6	1.1	< 0.3	< 0.3	< 0.3	< 0.3	< 0.1	< 0.3
Compost 5	Yes	0.6	1.2	< 0.3	< 0.3	< 0.3	< 0.3	< 0.1	< 0.3
Treated wood 1	No	< 0,3	< 0,2	n.a.	n.a.	n.a.	n.a.	n.a.	n.a.
Treated wood 2	No	1.8	12.5	n.a.	n.a.	n.a.	n.a.	n.a.	n.a.
Treated wood 3	Yes	< 0,3	11.1	n.a.	n.a.	n.a.	n.a.	n.a.	n.a.
Treated wood 4	No	1.0	6.7	n.a.	n.a.	n.a.	n.a.	n.a.	n.a.
Treated wood 5	No	0.3	0.6	n.a.	n.a.	n.a.	n.a.	n.a.	n.a.
Treated wood 6	No	0.6	1.9	n.a.	n.a.	n.a.	n.a.	n.a.	n.a.
Treated wood 7	No	< 0,3	< 0,2	n.a.	n.a.	n.a.	n.a.	n.a.	n.a.
Treated wood 8	No	0.5	1.2	n.a.	n.a.	n.a.	n.a.	n.a.	n.a.
Treated wood 9	No	< 0,3	1.1	n.a.	n.a.	n.a.	n.a.	n.a.	n.a.
Treated wood 10	No	< 0,3	< 0,2	n.a.	n.a.	n.a.	n.a.	n.a.	n.a.
Treated wood 11	No	< 0,3	7.7	n.a.	n.a.	n.a.	n.a.	n.a.	n.a.
Treated wood 12	No	< 0,3	1.8	n.a.	n.a.	n.a.	n.a.	n.a.	n.a.
Treated wood 13	Yes	0.5	0.8	n.a.	n.a.	n.a.	n.a.	n.a.	n.a.
Treated wood 14	No	n.a.	n.a.	n.a.	n.a.	n.a.	n.a.	n.a.	n.a.

*Heat map colouring is used to highlight higher concentrations of azole fungicides and those close to or higher than a tenth of the MIC for *A. fumigatus* are indicated in bold font. TBZ=tebuconazole, PPZ=propiconazole, MTZ=metconazole, EPZ=epoxiconazole, DFZ=difenoconazole, PTZ-S =prothioconazole-desthio, PBZ=paclobutrazole, MFZ=mefentrifluconazole.

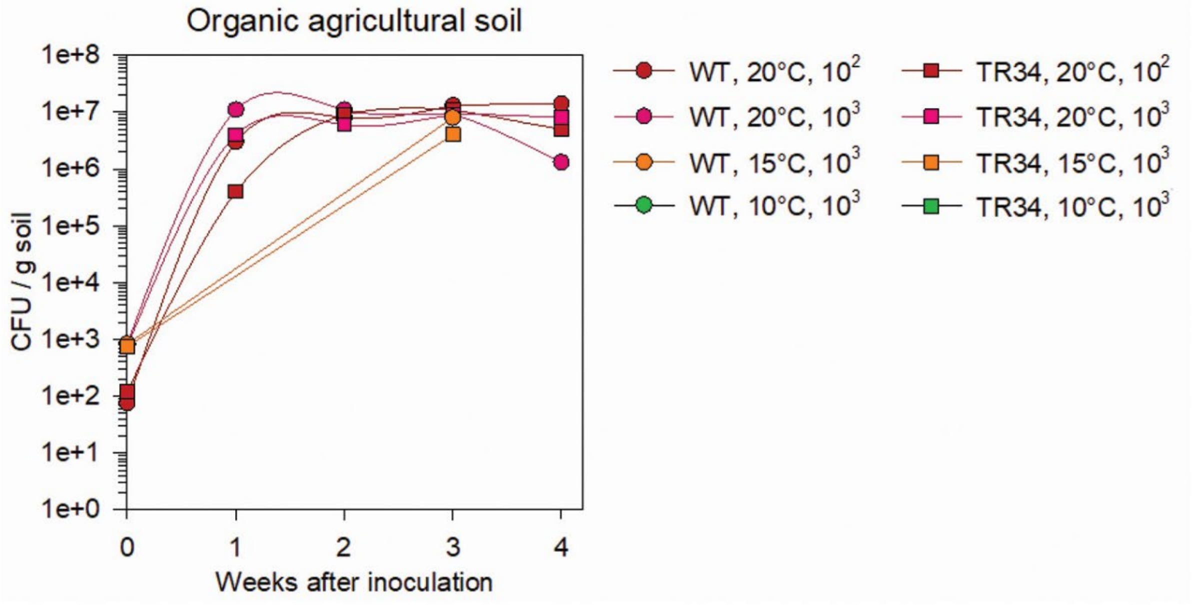

Appendix Figure 1. Test of growth conditions in microcosmos settings. The growth of *A. fumigatus* wild-type (WT) and TR_{34}/L98H (TR34) were measured by CFU counting. Samples incubated at 20°C were followed by weekly CFU counting for four weeks and two different inoculum sizes (10^2 and 10^3 CFU) were investigated. Samples incubated at 10°C or 15°C were only investigated three weeks after inoculation with 10^3 CFU, at which time no growth was found in samples incubated at 10 °C.

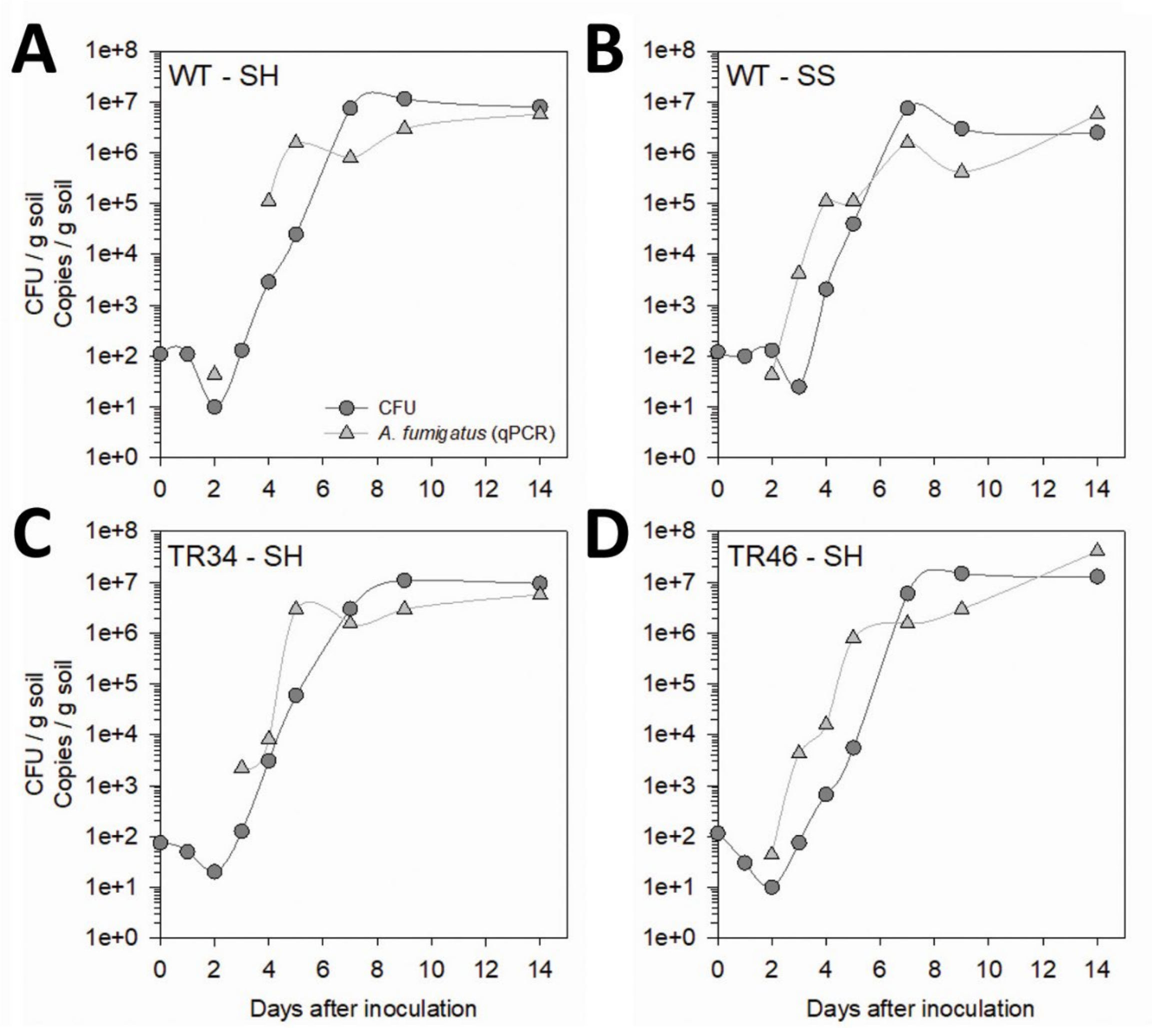

Appendix Figure 2. Growth kinetics of *A. fumigatus* wild-type (WT), TR_{34}/L98H (TR34) and TR_{46}/Y121F/T289A (TR46) in an organic rich soil (SH) and a sandy soil (SS). Quantification by CFU counting (dark grey line) and by qPCR (light grey) based detection of *A. fumigatus* (n=1) was compared. Early time points and the time point for the wild-type in the organic rich soil at day 3 were below the detection limit when analysed by qPCR.

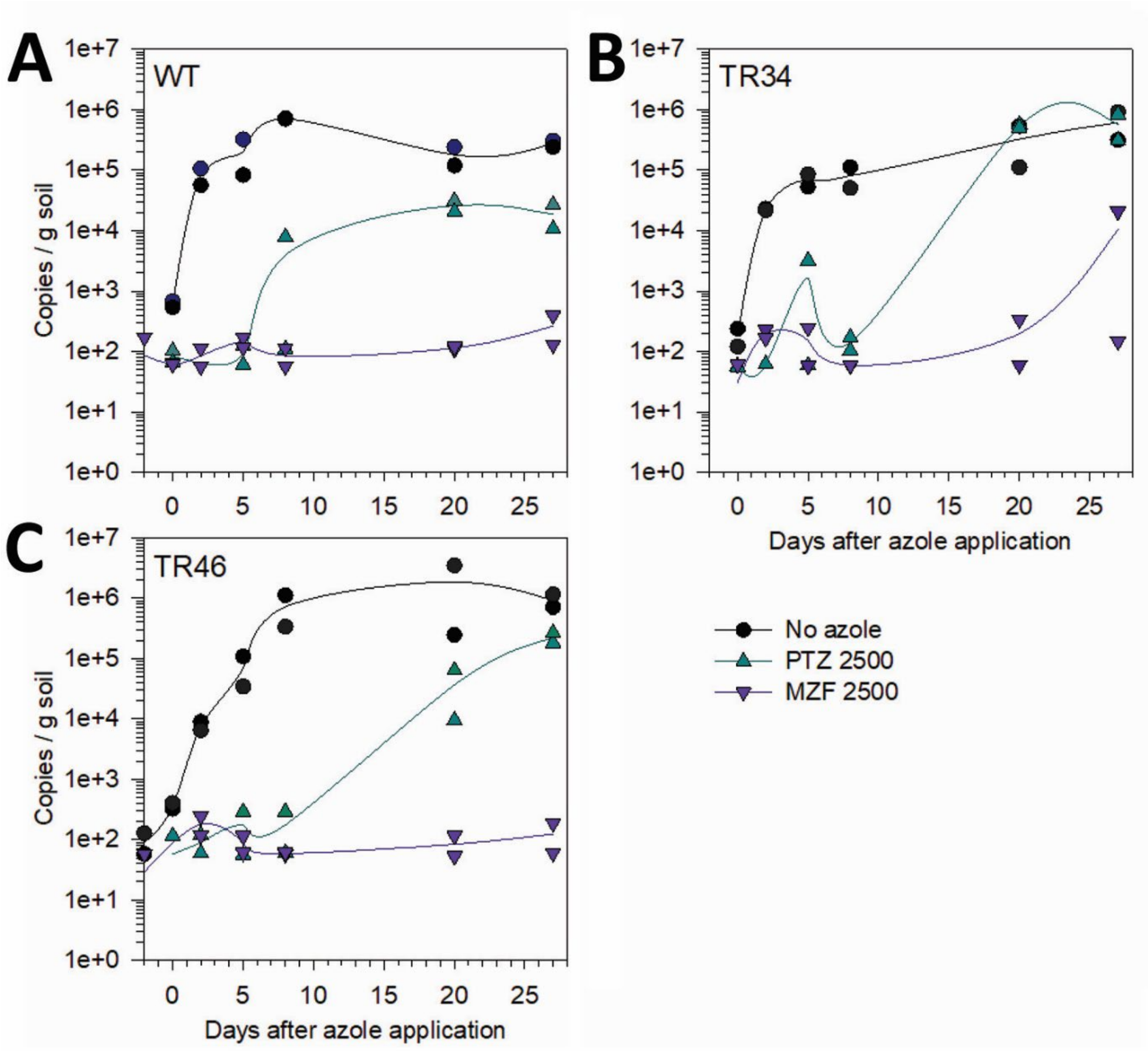

Appendix Figure 3. Selective pressure of prothioconazole (PTZ) and mefentrifluconazole (MZF) on *A. fumigatus* wild-type (WT), TR_{34}/L98H (TR34) and TR_{46}/Y121F/T289A (TR46) in sandy soil (n=2). The microcosmos were treated with 100 µL 2500 mg/L prothioconazole or mefentrifluconazole two days after inoculation. The symbols show each replicate and the line shows the average values of the duplicates. Untreated samples: black, samples treated with prothioconazole: blue, samples treated with mefentrifluconazole: purple. Growth was quantified by measuring copies of the *cyp51A* promoter region by ddPCR. The missing data points at day -2, 0 or 2 after azole application were due to lack of detection of the *cyp51A* gene in the sample.

References

1. Mellado E, Diaz-Guerra TM, Cuenca-Estrella M, Rodriguez-Tudela JL. Identification of two different 14-alpha sterol demethylase-related genes (*cyp51A* and *cyp51B*) in *Aspergillus fumigatus* and other *Aspergillus* species. J Clin Microbiol. 2001;39:2431–8. PubMed https://doi.org/10.1128/JCM.39.7.2431-2438.2001

2. Mortensen KL, Jensen RH, Johansen HK, Skov M, Pressler T, Howard SJ, et al. *Aspergillus* species and other molds in respiratory samples from patients with cystic fibrosis: a laboratory-based study with focus on *Aspergillus fumigatus* azole resistance. J Clin Microbiol. 2011;49:2243–51. PubMed https://doi.org/10.1128/JCM.00213-11

3. de Valk HA, Meis JFGM, Curfs IM, Muehlethaler K, Mouton JW, Klaassen CH. Use of a novel panel of nine short tandem repeats for exact and high-resolution fingerprinting of Aspergillus fumigatus isolates. J Clin Microbiol. 2005;43:4112–20. PubMed https://doi.org/10.1128/JCM.43.8.4112-4120.2005
